# Shifting Epidemiology and Antifungal Susceptibility Patterns of Clinical Fungal Isolates in an Intensive Care Unit (ICU) from Bucharest, Romania: A Retrospective Observational Study

**DOI:** 10.3390/antibiotics15050440

**Published:** 2026-04-29

**Authors:** Madalina (Preda) Solomon, Beatrice Mahler, Oana Popescu, Lia-Mara Ditu, Irina Gheorghe-Barbu, Laura Ioana Chivu, Loredana Sabina Cornelia Manolescu

**Affiliations:** 1 Department of Microbiology, Parasitology and Virology, Faculty of Nursing, Carol Davila University of Medicine and Pharmacy, 020021 Bucharest, Romania; madalina.preda@umfcd.ro (M.S.); loredana.manolescu@umfcd.ro (L.S.C.M.); 2 Clinical Laboratory of Medical Microbiology, Marius Nasta Institute of Pneumology, 050159 Bucharest, Romania; 3 Cardiothoracic Department, Faculty of Medicine, Carol Davila University of Medicine and Pharmacy, 020956 Bucharest, Romania; beatrice.mahler@umfcd.ro; 4 Pneumology Department, Marius Nasta Institute of Pneumology, 050159 Bucharest, Romania; 5 National Reference Laboratory of Tuberculosis, Marius Nasta Institute of Pneumology, 050159 Bucharest, Romania; oana.popescu@marius-nasta.ro; 6 Department of Botany and Microbiology, Faculty of Biology, University of Bucharest, Portocalilor 1-3, 060101 Bucharest, Romania; irina.gheorghe@bio.unibuc.ro; 7 MICROGEN Research Centre, Faculty of Biology, University of Bucharest, Portocalilor 1-3, 060101 Bucharest, Romania; 8 Research Institute of the University of Bucharest, University of Bucharest, 050663 Bucharest, Romania; 9 Department of Pathophysiology, Faculty of Nursing, Carol Davila University of Medicine and Pharmacy, 020021 Bucharest, Romania; laura.chivu@umfcd.ro

**Keywords:** Candida, antifungal resistance, azoles, echinocandins, multidrug resistance

## Abstract

Background: Antifungal resistance among Candida species represents a growing clinical challenge, particularly in the context of increasing prevalence of non-albicans species. Methods: We conducted a retrospective analysis of 747 fungal isolates collected between 2021 and 2026, evaluating species distribution, antifungal susceptibility profiles, minimum inhibitory concentration (MIC) patterns, and temporal trends in resistance. Results: Non-albicans Candida species accounted for 67.5% of isolates, exceeding the proportion of *Candida albicans*. Fluconazole and flucytosine exhibited the highest resistance rates (16.1% and 17.4%, respectively), while amphotericin B showed the highest susceptibility rate (82.1%). MIC analysis revealed elevated MIC90 values for azoles in *Candida glabrata* and *Candida krusei*, consistent with reduced susceptibility. A significant association between antifungal agent and susceptibility profile was observed (χ^2^ = 33.81, *p* < 0.001; Cramér’s V = 0.205). Temporal analysis demonstrated fluctuating resistance patterns rather than a consistent increase over time. Multidrug resistance was uncommon (2.5%), although non-susceptibility to multiple agents was more frequent (20.3%). Conclusions: The study highlights substantial variability in antifungal susceptibility across Candida species and antifungal agents, emphasizing the importance of continuous surveillance and species-specific treatment strategies.

## 1. Introduction

Fungal infections caused by Candida species represent a major source of morbidity and mortality, particularly among critically ill patients admitted to intensive care units (ICUs). This population is especially vulnerable due to multiple predisposing factors, including invasive procedures, prolonged hospitalization, and exposure to broad-spectrum antimicrobial therapy [1,2,3]. Invasive candidiasis remains one of the most important healthcare-associated fungal infections in critically ill patients, with persistently high attributable mortality and major diagnostic challenges in ICU settings [1,2,3].

Traditionally, *Candida albicans* (*C. albicans*) has been considered the predominant etiological agent of candidiasis. However, a progressive shift toward non-albicans Candida species has been increasingly reported in recent years, including in ICU cohorts and hospital-based surveillance studies [4,5,6,7]. Species such as *Nakaseomyces glabratus* (formerly *Candida glabrata*), *Candida parapsilosis*, *Candida tropicalis*, and *Pichia kudriavzevii* (formerly *Candida krusei*) are now recognized as clinically important pathogens because of their distinct antifungal susceptibility profiles, capacity for persistence in healthcare environments, and, in some cases, intrinsic or acquired resistance to commonly used antifungal agents [4,5,6,7,8].

This epidemiological shift has important therapeutic implications, especially in ICU patients, where invasive devices, prior antimicrobial exposure, prolonged hospitalization, and empiric antifungal treatment may all contribute to species redistribution and antifungal selection pressure [2,3,5,6,9]. Recent surveillance data from Europe and recent hospital-based susceptibility studies have further emphasized marked inter-center variability in Candida epidemiology and resistance phenotypes, underscoring the importance of local data for guiding empirical and targeted therapy [5,6,10].

Antifungal resistance in Candida species involves multiple mechanisms, including drug target alterations, increased efflux pump activity, and activation of stress response pathways [9,11]. These processes are driven by genetic and epigenetic changes, contributing to reduced susceptibility and treatment failure [11,12].

Antifungal resistance has emerged as a major global concern, driven by increased antifungal use, prolonged treatment courses, and selective pressure within healthcare environments. Resistance to azoles, particularly fluconazole, is widely documented and is commonly associated with alterations in ergosterol biosynthesis pathways and/or increased efflux activity [8,13]. In parallel, echinocandin resistance, although still less frequent overall, is increasingly reported, particularly among *C. glabrata*, and is mainly associated with mutations in the FKS genes [12,14]. Amphotericin B remains a broad-spectrum antifungal agent with relatively preserved activity against most Candida species, although its clinical utility is limited by toxicity [15]. Flucytosine remains useful in selected clinical settings, particularly in combination regimens, but interpretation of its activity is more complex because resistance may emerge rapidly, and species-specific MIC distributions can be highly variable [16].

In ICU-centered cohorts, surveillance studies are especially important because they can capture both changes in species prevalence and shifts in antifungal susceptibility over time [16,17]. In addition to categorical susceptibility interpretations, the analysis of minimum inhibitory concentration (MIC) distributions, including MIC50 and MIC90 values, provides a more detailed view of antifungal activity and may help identify the emergence of resistant subpopulations before major epidemiological changes become evident [16,17]. In critically ill patients, this approach is increasingly relevant given the growing interest in fungal colonization dynamics, early detection strategies, and hospital ecology as contributors to invasive disease [17,18].

In this context, the present study provides real-world data from a tertiary care hospital specialized in respiratory diseases and focuses on fungal isolates recovered from ICU patients over a five-year and three-month period. The aim was to characterize species distribution, antifungal susceptibility patterns, MIC distributions, and temporal resistance trends in order to better define the local epidemiology of Candida infections in a critically ill population.

## 2. Results

### 2.1. Study Population and Temporal Distribution

A total of 747 fungal isolates from 725 unique patients were included in the analysis. All isolates were obtained from patients admitted to intensive care units (ICUs), reflecting a critically ill population at high risk for invasive fungal infections. Basic demographic data, including age and sex, were available from routine laboratory records, indicating a heterogeneous patient population within the ICU setting. The isolates were collected between 2021 and 2026, although the yearly distribution was uneven. The highest number of isolates was recorded in 2023 (*n* = 189; 25.3%), followed by 2024 (*n* = 176; 23.6%) and 2021 (*n* = 170; 22.8%). Lower numbers were observed in 2025 (*n* = 112; 15.0%) and 2022 (*n* = 82; 11.0%), whereas only 18 isolates (2.4%) were available for 2026, limiting the reliability of year-specific estimates for that period.

### 2.2. Species Distribution and Changes over Time

The dataset was dominated by *Candida* spp. Overall, *C. albicans* was the most frequent species, accounting for 243/747 isolates (32.5%). This was followed by *C. glabrata* (135/747; 18.1%), *C. parapsilosis* (84/747; 11.2%), *C. krusei* (69/747; 9.2%), and *C. tropicalis* (62/747; 8.3%). Together, these five species represented 593/747 isolates (79.4%).

Other species were less frequent but still clinically relevant, including *Candidozyma auris* (formerly known as *Candida auris*, *C. auris*) (38 isolates; 5.1%), *Clavispora lusitaniae* (*Candida lusitaniae*, *C. lusitaniae*) (31; 4.1%), *Kluyveromyces marxianus* (formerly *Candida kefyr*, *C. kefyr* (25; 3.3%), *Candida dubliniensis* (15; 2.0%), and *Papiliotrema laurentii* (formerly Cryptococcus laurentii, *C. laurentii*) (10; 1.3%) (Table 1). The remaining species each accounted for <1% of the total dataset.

Importantly, non-*albicans Candida* species collectively represented 504/747 isolates (67.5%), exceeding the proportion of *C. albicans*. This indicates a shift from the traditional predominance of *C. albicans*.

**Figure 1:**
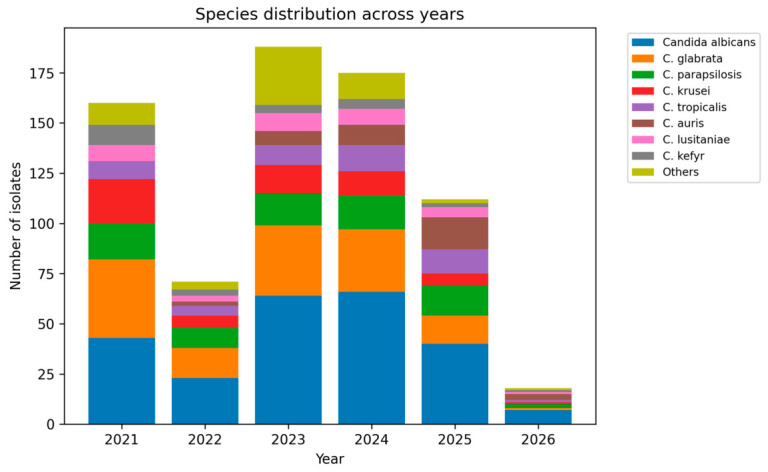
Species distribution across years (2021–2026). Stacked bar chart showing the annual distribution of major *Candida* species. The analysis includes *Candida albicans*, *C. glabrata*, *C. parapsilosis*, *C. krusei*, *C. tropicalis*, *C. auris*, *C. lusitaniae*, and *C. kefyr*, while less frequent species are grouped as “Others”. The figure illustrates temporal variability in species distribution, including the emergence and increase in *C. auris* in later years.

The relative contribution of major species varied across years (Figure 1).

The proportion of *C. albicans* increased from 25.3% in 2021 to 33.9% in 2023, 37.5% in 2024, and 35.7% in 2025. By contrast, *C. glabrata* showed a gradual decline, from 22.9% in 2021 to 18.5% in 2023, 17.6% in 2024, and 12.5% in 2025.

*C. parapsilosis* remained relatively stable, fluctuating between 9.5% and 13.4% depending on the year. *C. krusei* was more frequent in the early years (12.9% in 2021 and 14.6% in 2022) and declined thereafter (9.0% in 2023, 5.7% in 2024, and 6.2% in 2025). In contrast, *C. tropicalis* increased over time, rising from 6.5% in 2021 and 5.8% in 2023 to 10.2% in 2024 and 14.3% in 2025.

These data indicate that the dataset was not static over time and that the fungal ecology shifted during the study period, particularly with regard to *C. albicans*, *C. glabrata*, and *C. tropicalis*.

### 2.3. Distribution by Specimen Source

**Figure 2:**
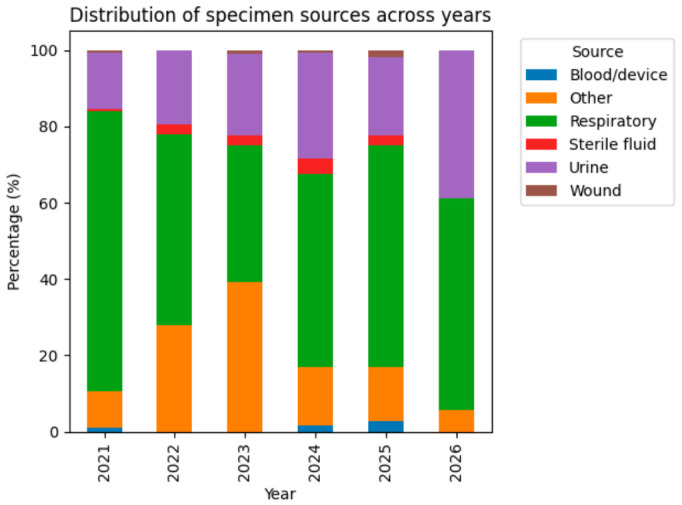
Distribution of specimen sources across years (2021–2026). The figure illustrates the relative proportion of major specimen types contributing to fungal isolation. Respiratory samples consistently represented the predominant source, followed by urinary samples, with minor contributions from other specimen types. No major temporal shifts in specimen distribution were observed.

All samples were collected at the time of ICU admission, reflecting the clinical context of critically ill patients. The distribution of isolates by specimen source showed a marked predominance of respiratory samples, which accounted for 400/747 isolates (53.5%)**.** Urinary samples represented 165/747 isolates (22.1%). Smaller groups included wound/soft tissue samples (29; 3.9%), sterile fluid/CNS samples (19; 2.5%), blood/device-related samples (14; 1.9%), and other sources (62; 8.3%) (Figure 2).

Importantly, the relative distribution of specimen sources remained broadly consistent across the study period, with respiratory samples predominating each year, followed by urinary samples. No major shifts in the proportion of specimen types were observed over time, suggesting that the observed temporal changes in species distribution are unlikely to be driven by variations in sampling patterns.

Although minor year-to-year fluctuations were noted in the absolute number of isolates per specimen type, the overall ranking of specimen sources remained stable, with respiratory samples consistently representing the largest proportion of isolates throughout the study period.

When species and source were examined together, *C. albicans* was the most frequent species in both respiratory (140 isolates) and urinary (51 isolates) samples. *C. glabrata* was also highly represented in respiratory material and remained common in urine. *C. tropicalis* showed a marked concentration in respiratory samples (35 isolates) compared with urinary samples (11 isolates). These findings indicate a high representation of respiratory samples in the dataset, which may include both infection and colonization; however, given that samples were obtained from symptomatic ICU patients, colonization is likely limited and should be interpreted with caution.

Taken together, these findings suggest that the observed shifts in species distribution occurred in the context of relatively stable sampling patterns, supporting a potential epidemiological shift rather than a bias related to specimen source distribution.

### 2.4. Overall Antifungal Susceptibility Profile

Analysis of antifungal susceptibility demonstrated heterogeneous resistance patterns across agents (Table 2).

Among 385 isolates with interpretable fluconazole results, 305 (79.2%) were susceptible, 18 (4.7%) were intermediate, and 62 (16.1%) were resistant. The overall fluconazole resistance rate was therefore 16.1%, while the non-susceptibility rate (I + R) reached 20.8%.

For voriconazole, 426 isolates had interpretable results. Of these, 317 (74.4%) were susceptible, 92 (21.6%) were intermediate, and 17 (4.0%) were resistant. Although the resistance rate was low (4.0%), the non-susceptibility rate was substantially higher (25.6%) because of the large intermediate category.

Among 249 interpretable caspofungin results, 194 (77.9%) were susceptible, 45 (18.1%) were intermediate, and 10 (4.0%) were resistant. The resistance rate was 4.0%, whereas the non-susceptibility rate was 22.1%.

For micafungin, 424 isolates were interpretable. Of these, 297 (70.0%) were susceptible, 116 (27.4%) were intermediate, and 11 (2.6%) were resistant. Micafungin exhibited the lowest resistance rate among the six agents (2.6%), but the highest non-susceptibility rate (30.0%) because of the large proportion of intermediate results.

Amphotericin B yielded 588 interpretable results, of which 483 (82.1%) were susceptible, 64 (10.9%) were intermediate, and 41 (7.0%) were resistant. Despite being the best-covered antifungal in the dataset, amphotericin B still showed measurable resistance.

For flucytosine, 236 isolates had interpretable results. 182 (77.1%) were susceptible, 13 (5.5%) were intermediate, and 41 (17.4%) were resistant. Flucytosine, therefore, exhibited the highest resistance rate in the entire dataset (17.4%).

The distribution of minimum inhibitory concentrations (MICs) across Candida species revealed notable interspecies variability in antifungal susceptibility (Table 3). MIC50 and MIC90 values demonstrated that while most species remained susceptible to echinocandins and amphotericin B, elevated MIC90 values were observed for azoles in specific species, particularly *C. glabrata* and *C. krusei*, indicating reduced susceptibility and known resistance patterns. *C. auris* isolates (*n* = 5 with available MIC data) showed elevated MIC values for fluconazole (MIC50 = 64 mg/L) and amphotericin B (MIC50 = 8 mg/L), while micafungin MIC values remained low (MIC50 = 0.12 mg/L). *C. auris* isolates showed consistently elevated MIC values for amphotericin B and fluconazole, suggesting reduced susceptibility (Table 3).

**Figure 3:**
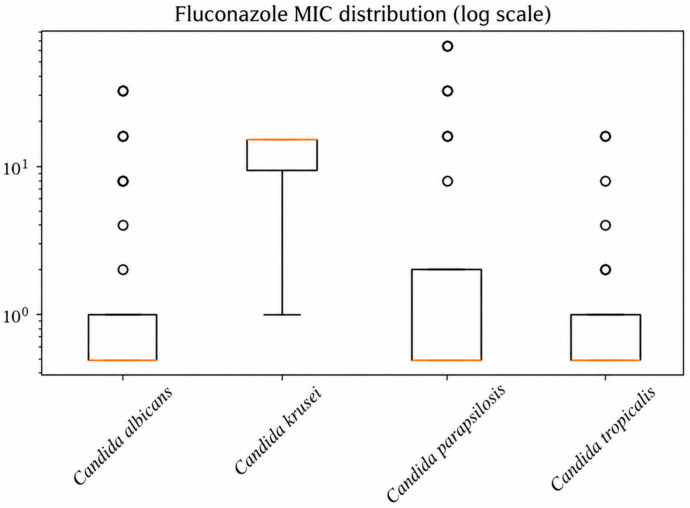
Distribution of fluconazole MIC values across Candida species. The boxplots demonstrate interspecies variability, with higher MIC values observed in *Candida krusei*, consistent with reduced susceptibility to fluconazole.

The distribution of fluconazole minimum inhibitory concentrations (MICs) across Candida species is presented in Figure 3, demonstrating variability in susceptibility and higher MIC values among non-albicans species.

To further explore differences between antifungal agents, comparative statistical analyses were performed.

### 2.5. Comparative Analysis of Resistance Across Antifungals

The distribution of susceptibility outcomes, categorized as susceptible, intermediate, and resistant, revealed notable variability between antifungal classes. Amphotericin B demonstrated the highest proportion of susceptible isolates, accounting for approximately 82.1% of cases, followed by voriconazole and fluconazole, both exhibiting susceptibility rates of approximately 55–56%. In contrast, micafungin and caspofungin showed moderate susceptibility profiles, while flucytosine presented the lowest proportion of susceptible isolates and a relatively higher rate of resistance.

When direct resistance rates were compared, the highest values were observed for flucytosine (17.4%) and fluconazole (16.1%). Intermediate susceptibility was observed for amphotericin B (7.0%), whereas voriconazole, caspofungin, and micafungin all remained below 5% resistance.

However, when non-susceptibility was used as the comparator, the ranking changed. Micafungin showed the highest non-susceptibility rate (30.0%), followed by voriconazole (25.6%), flucytosine (22.9%), caspofungin (22.1%), fluconazole (20.8%), and amphotericin B (17.9%). Thus, the dataset showed a clear divergence between strict resistance and broader reduced susceptibility.

A global comparison of susceptibility distributions using the chi-square test indicated statistically significant differences among antifungal agents (χ^2^ = 33.81, df = 10, *p* < 0.001), confirming that the observed variation is unlikely to be due to random chance. The strength of this association, assessed using Cramér’s V, was 0.205, suggesting a small-to-moderate effect size.

To further explore the source of this heterogeneity, standardized residual analysis was performed (Table 4). This revealed that fluconazole exhibited a significantly higher-than-expected resistance rate (residuals > 2) of resistant isolates (residual = +2.36) alongside a deficit of intermediate cases (−2.27), while flucytosine also demonstrated an excess of resistance (residual = +2.15), suggesting a non-uniform distribution of resistance across antifungal agents. In contrast, micafungin showed a significant excess of intermediate susceptibility (residual = +2.59), suggesting a distinct susceptibility pattern compared to the other agents, which may indicate emerging resistance or dose-dependent activity. Amphotericin B demonstrated a stable susceptibility profile without significant deviations, supporting its role as a highly effective antifungal agent.

Pairwise comparisons focusing on susceptible versus resistant categories did not reveal statistically significant differences after accounting for multiple comparisons; however, several comparisons approached statistical significance, including fluconazole versus micafungin (*p* ≈ 0.056) and micafungin versus flucytosine (*p* ≈ 0.055). These findings suggest the presence of biologically relevant trends that may not reach statistical significance due to sample size limitations.

Odds ratio analysis demonstrated that fluconazole and flucytosine exhibited higher odds of resistance compared to amphotericin B (OR = 2.53 and 2.74, respectively). Micafungin showed a lower odds of resistance (OR = 0.51), suggesting a potentially favorable susceptibility profile.

Amphotericin B was used as the reference agent for odds ratio calculations due to its broad-spectrum activity and consistently low resistance rates, making it a suitable benchmark for antifungal susceptibility comparisons. Although these differences did not reach statistical significance, they provide additional support for differential antifungal efficacy profiles.

### 2.6. Species-Specific Susceptibility Patterns

Among *C. albicans* isolates, fluconazole yielded 218 interpretable results, with 36 resistant isolates, corresponding to a resistance rate of 16.5%. Voriconazole resistance was lower, 6/157 isolates (3.8%), although the non-susceptibility rate was much higher because 81/157 isolates were intermediate. For micafungin, resistance remained low (4/174; 2.3%), but non-susceptibility reached 52.3% because most non-susceptibility isolates were intermediate. Amphotericin B resistance in *C. albicans* was 24/216 (11.1%), which was higher than the overall amphotericin B resistance rate of 7.0%, although this difference should be interpreted cautiously given differences in sample sizes and species composition (Table 5).

*C. glabrata* showed a highly distinctive pattern. For fluconazole, there were 14 interpretable results; none were categorized as susceptible or resistant; all isolates were classified as intermediate; all 14/14 (100%) were intermediate. Thus, the resistance rate was 0%, but the non-susceptibility rate was 100%. For caspofungin, *C. glabrata* showed 9 resistant isolates among 74 tested (12.2%), with an additional 27 intermediate isolates, resulting in 48.6% non-susceptibility. Micafungin resistance was 4/97 (4.1%), with a non-susceptibility rate of 34.0%. This species, therefore, contributed disproportionately to the echinocandin non-susceptibility burden.

*C. parapsilosis* remained largely susceptible overall, but important exceptions were observed. Fluconazole resistance reached 12/80 (15.0%), voriconazole resistance was 6/75 (8.0%), and amphotericin B resistance was 4/79 (5.1%). By contrast, no resistance was observed for caspofungin in the 31 tested isolates, and only 1/80 isolates (1.2%) were resistant to micafungin.

For *C. tropicalis*, fluconazole resistance was 7/54 (13.0%), voriconazole resistance 2/53 (3.8%), and amphotericin B resistance 2/54 (3.7%). The most notable finding for this species was flucytosine resistance: 6/16 tested isolates (37.5%) were resistant, with an additional intermediate result, resulting in 43.8% non-susceptibility.

*C. krusei* showed the expected intrinsic pattern for fluconazole: 4/4 tested isolates (100%) were resistant. In addition, flucytosine resistance was 26/26 (100%). No resistant isolates were recorded for voriconazole, caspofungin, or micafungin, although 17/37 caspofungin-tested isolates were intermediate, resulting in 45.9% non-susceptibility. Amphotericin B resistance was 3/59 (5.1%).

Although 38 isolates of *C. auris* were identified, no interpretable susceptibility results were available. Antifungal susceptibility testing, including MIC determination, was performed only for a limited number of isolates; however, the number of tested isolates was insufficient for meaningful statistical analysis. In addition, routine testing was not consistently implemented due to the lack of standardized interpretive guidelines and limitations of the laboratory system.

### 2.7. Annual Changes in Resistance

Temporal analysis of resistance showed fluctuations across years rather than a consistent increasing trend (Table 6).

Year-specific resistance rates varied across drugs. For amphotericin B, resistance increased from 0.6% in 2021 to 4.5% in 2022, peaked at 12.9% in 2023, and then declined to 6.8% in 2024 before rising slightly to 7.9% in 2025. Voriconazole resistance was 1.3% in 2021, increased to 8.7% in 2023, remained moderate in 2024 (6%), and again rose in 2025 (8.6%).

Micafungin resistance was absent in 2021 and 2022, but appeared in 2023 (1.0%), increased in 2024 (6.6%), and reached 8.3% in 2025. Caspofungin resistance remained low and inconsistent over time, fluctuating between 0% and 5.6% depending on the year. Because only 18 isolates were available for 2026, yearly rates for that year were not considered stable enough for interpretation, and the markedly high amphotericin B resistance observed (80.0%) should therefore be interpreted with extreme caution.

**Figure 4:**
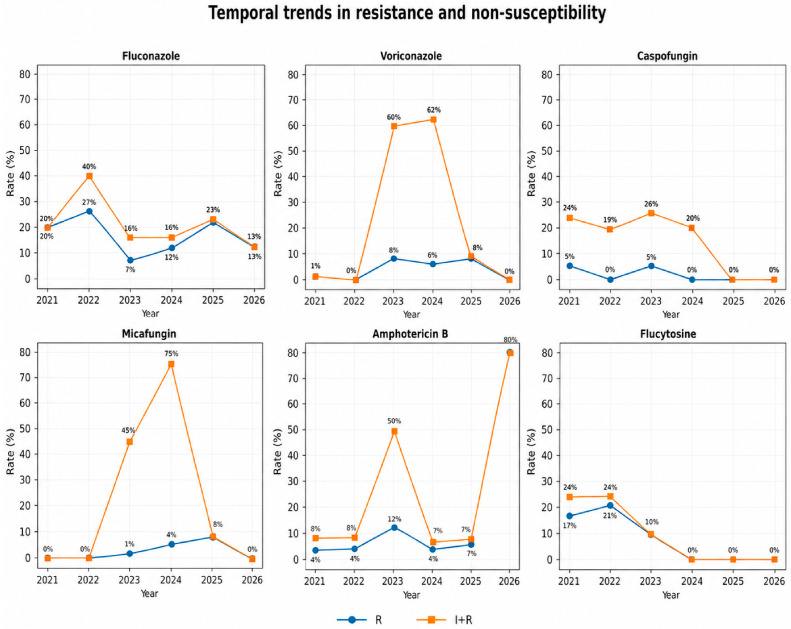
Temporal trends in resistance (R) and non-susceptibility (I + R) rates across antifungal agents from 2021 to 2026, demonstrating variability in susceptibility patterns over time.

Overall, the temporal data suggest fluctuation rather than a monotonic rise across all agents, but they also show that certain antifungals—especially micafungin and voriconazole—displayed higher resistance rates in later years than in the earliest part of the study period (Figure 4).

**Figure 5:**
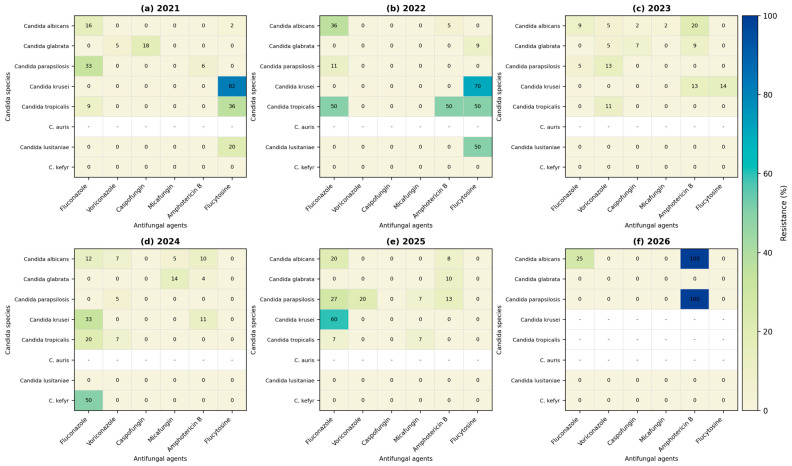
Yearly heatmaps of antifungal resistance rates across Candida species (2021–2026). Panels represent annual resistance profiles for each antifungal agent and species: (**a**) 2021, (**b**) 2022, (**c**) 2023, (**d**) 2024, (**e**) 2025, and (**f**) 2026. Color intensity reflects resistance rates (%), with higher values indicated by warmer colors. The heatmaps illustrate temporal variability in resistance patterns across both antifungal agents and Candida species, with notable increases in resistance observed for fluconazole in multiple species. Cells marked with “-” indicate either that no isolates were identified in the respective year or that no interpretive susceptibility results were available according to current guidelines.

Yearly resistance patterns across Candida species are presented as heatmaps in Figure 5. These visualizations highlight temporal fluctuations in antifungal resistance rates, revealing dynamic changes across both species and antifungal classes. Intermittent increases in fluconazole resistance are observed across multiple species over time, while *C. krusei* consistently exhibits high resistance levels, in line with its known intrinsic resistance profile. Variability in resistance patterns is also evident among echinocandins and amphotericin B, suggesting evolving susceptibility trends across the study period.

### 2.8. Multidrug Resistance

At the isolate level, 19/747 isolates (2.5%) were resistant to at least two antifungals. In contrast, 152/747 isolates (20.3%) were non-susceptible to at least two agents when both intermediate and resistant categories were considered. These findings indicate that while strict multidrug resistance remains rare, reduced susceptibility across multiple antifungal classes is relatively common (Table 7).

## 3. Discussion

All samples were collected at the time of ICU admission, suggesting that the isolates do not reflect infections acquired during the current hospitalization. However, due to the lack of data on prior healthcare exposure, it was not possible to definitively classify infections as community-acquired or healthcare-associated. In addition, the retrospective laboratory-based design did not allow differentiation between primary and secondary infections. The predominance of respiratory samples may reflect colonization or infection in mechanically ventilated patients, which is common in ICU settings.

The present study provides a comprehensive analysis of antifungal susceptibility patterns across Candida species, confirming a predominance of non-albicans Candida species. This finding is consistent with multiple large-scale surveillance studies, including the SENTRY Antimicrobial Surveillance Program, which has documented a global increase in non-albicans species over recent decades [19,20]. In particular, the increased prevalence of *C. glabrata* and *C. parapsilosis* observed in our dataset aligns with trends reported in both European and North American cohorts [19,21]. Furthermore, the predominance of non-albicans Candida species in our ICU-based cohort supports recent observations that critically ill populations represent a distinct epidemiological niche characterized by increased antifungal exposure, invasive procedures, and prolonged hospitalization [5,6,22].

*C. albicans* is traditionally considered the most virulent Candida species, largely due to its ability to undergo morphological transitions (yeast-to-hyphae switching), invade host tissues, and evade immune responses [23,24,25]. In contrast, non-albicans Candida species generally exhibit lower intrinsic virulence but have emerged as important pathogens in hospitalized and critically ill patients, particularly in intensive care settings [23,24,25]. Their increasing clinical relevance is largely associated with antifungal resistance, biofilm formation, and persistence in healthcare environments [23,24,25].

In the present study, the predominance of non-albicans Candida species, together with the observed variability in antifungal susceptibility, particularly the higher resistance rates to fluconazole, supports this shift in epidemiology. These findings are clinically relevant, as non-albicans Candida species often require alternative therapeutic approaches due to reduced susceptibility to commonly used antifungal agents. Therefore, accurate species identification is essential for guiding antifungal therapy and optimizing clinical outcomes in ICU patients. This shift toward non-*albicans Candida* species may partly explain the increased antifungal resistance observed in ICU settings.

The observed resistance rates for fluconazole (16.1%) are comparable to those reported in recent multicenter studies, where resistance rates typically range between 10% and 20%, depending on geographic region and patient population [20,26,27,28]. The elevated MIC90 values observed in *C. glabrata* and *C. krusei* further support the well-established reduced susceptibility of these species to azoles. *C. krusei* is known to exhibit intrinsic resistance to fluconazole, while *C. glabrata* often displays dose-dependent susceptibility or resistance mediated by efflux pump overexpression and alterations in ergosterol biosynthesis pathways [20,26,27]. Recent ICU-focused studies have also shown that azole resistance may be more pronounced in critically ill patients, likely reflecting cumulative antifungal exposure and colonization pressure.

In contrast, voriconazole resistance remained relatively low in our dataset, which is consistent with previous studies demonstrating retained activity of second-generation azoles against many Candida species [29,30]. However, the high proportion of intermediate isolates observed in this study suggests that reduced susceptibility may be more widespread than resistance alone would indicate. This finding is particularly relevant in the context of EUCAST definitions, where the intermediate category corresponds to “susceptible, increased exposure,” highlighting the need for optimized dosing strategies in critically ill patients [31,32,33,34].

Echinocandins, including caspofungin and micafungin, demonstrated low resistance rates, supporting their role as first-line agents in invasive candidiasis [26,29]. Nevertheless, the relatively high proportion of intermediate susceptibility observed for micafungin in our dataset may reflect early shifts in susceptibility patterns. Emerging echinocandin resistance has been increasingly linked to mutations in the FKS genes, and even modest increases in MIC values may have clinical significance [29]. However, under EUCAST definitions, the intermediate category corresponds to “susceptible, increased exposure,” rather than true resistance. According to the most recent EUCAST clinical breakpoints (version 12.0), susceptibility should be interpreted in the context of pharmacokinetic/pharmacodynamic parameters, particularly in ICU patients, where altered drug exposure may influence treatment outcomes [34].

Amphotericin B exhibited the highest overall susceptibility rate, consistent with its broad-spectrum activity and historically low resistance rates [30]. However, the presence of measurable resistance in our dataset highlights that reduced susceptibility can still occur and should not be overlooked. Previous studies have also reported occasional increases in amphotericin B MIC values, particularly in patients with prolonged antifungal exposure, reinforcing the need for continued surveillance [30].

The temporal variability in antifungal resistance rates observed in this study is likely multifactorial. Changes in species distribution, particularly fluctuations in the proportion of non-albicans Candida species, may have influenced resistance patterns across different years. In addition, selective pressure associated with antifungal use in ICU settings may contribute to the emergence of resistant strains.

The absence of a consistent increasing or decreasing trend suggests that resistance dynamics are complex and influenced by local epidemiology. Moreover, variations in the number of isolates per year may also affect the observed resistance rates. The marked increase in amphotericin B resistance observed in 2026 should be interpreted with caution, as it is likely related to the small number of isolates analyzed in that year rather than a true epidemiological shift.

The statistical analysis further reinforces the presence of heterogeneity in susceptibility patterns across antifungal agents. The significant chi-square result indicates that susceptibility distributions differ across antifungal classes, while the moderate effect size suggests that these differences are clinically meaningful but not extreme. Residual analysis identified fluconazole and flucytosine as major contributors to this variability, with higher-than-expected resistance rates. This approach allows identification of antifungal-specific deviations beyond simple proportion comparisons and has been increasingly applied in epidemiological studies to better characterize susceptibility datasets [31]. Temporal analysis in the present study revealed fluctuating resistance patterns rather than a consistent upward trend. This observation is consistent with findings from longitudinal surveillance studies, which have shown that antifungal resistance does not always increase linearly but may vary depending on antifungal usage patterns, infection control measures, and local epidemiology [19,32]. The apparent increase in resistance for certain agents, such as micafungin and voriconazole, in later years may reflect evolving selective pressures, although further investigation is needed to confirm these trends. Such variability is particularly relevant in ICU settings, where antimicrobial stewardship interventions and patient case-mix may significantly influence resistance dynamics [5].

Species-specific analysis revealed distinct susceptibility patterns consistent with known biological characteristics. *C. glabrata* demonstrated high non-susceptibility to azoles, while *C. krusei* exhibited intrinsic resistance to fluconazole. These findings highlight the importance of species identification in guiding antifungal therapy [21]. Additionally, *C. parapsilosis* showed relatively higher MIC values for echinocandins, attributed to naturally occurring differences in the glucan synthase target enzyme [29].

A limitation of the present study is the lack of detailed clinical correlation. Information regarding patient characteristics, including comorbidities and confirmed diagnosis of invasive infection, was not consistently available. However, all isolates were obtained from patients admitted to the intensive care unit of a tertiary care hospital specialized in respiratory diseases, and samples were collected only from patients with clinical signs and symptoms suggestive of infection. Therefore, although colonization cannot be fully excluded—particularly for respiratory samples—its contribution is expected to be relatively limited.

The high proportion of respiratory isolates represents an important limitation, as Candida species are known colonizers of the respiratory tract, particularly in critically ill patients. However, given that samples were obtained from symptomatic ICU patients, these findings are likely to reflect clinically relevant isolates rather than routine colonization. Previous studies have also emphasized that respiratory colonization may be associated with disease severity and may precede invasive infection in selected cases [17,33]. Multidrug resistance was relatively uncommon in this cohort when defined strictly as resistance to multiple agents. However, when intermediate susceptibility was included, a substantial proportion of isolates exhibited reduced susceptibility to more than one antifungal. This finding is clinically relevant, as intermediate susceptibility may compromise treatment efficacy, particularly in severe infections requiring optimal antifungal exposure. Similar observations have been reported in recent studies, highlighting the clinical significance of non-susceptibility phenotypes [29,31].

The relatively high proportion of *C. auris* observed in our study (approximately 5%) is noteworthy compared to reports from other European settings [25]. This finding may reflect local epidemiological characteristics, increased detection, or potential limitations related to species identification using automated systems. Although identification was performed using the Vitek 2 system under standard laboratory conditions with appropriate quality control measures, it is acknowledged that automated systems may have limitations in distinguishing closely related yeast species. Molecular confirmation was not systematically performed due to the retrospective design of the study, which represents a limitation. However, all *C. auris* isolates were cryopreserved and are currently included in an ongoing study aimed at further characterization using alternative susceptibility testing methods and genetic sequencing. These isolates are available for further research and collaborative studies upon reasonable request.

In critically ill patients, altered pharmacokinetics may significantly impact antifungal exposure, leading to suboptimal pharmacodynamic target attainment even when in vitro susceptibility results suggest adequate activity [35,36,37]. Marked pharmacokinetic variability in critically ill patients may lead to suboptimal antifungal exposure despite standard dosing, highlighting the importance of individualized dosing and therapeutic drug monitoring [38].

Increasing antifungal resistance among nosocomial Candida isolates further limits therapeutic options and may contribute to poorer clinical outcomes, emphasizing the need for continuous surveillance and improved treatment strategies [25,39].

Antifungal resistance in Candida species is driven by multiple molecular mechanisms that enable adaptation to antifungal exposure [11]. The most important include alterations of drug targets, increased drug efflux, and activation of cellular stress response pathways [11]. These mechanisms are frequently associated with genetic changes such as point mutations, chromosomal rearrangements (e.g., aneuploidy), and epigenetic regulation, reflecting the high genomic plasticity of fungal pathogens [11,40]. At the molecular level, azole resistance is commonly associated with mutations or overexpression of the ERG11 gene, which encodes the target enzyme involved in ergosterol biosynthesis, as well as increased activity of efflux pumps that reduce intracellular drug accumulation [11,40]. Resistance to echinocandins is primarily mediated by mutations in the FKS genes encoding β-(1,3)-glucan synthase, while additional adaptive responses include cell wall remodeling and stress-response signaling pathways [11,40]. Biofilm formation further contributes to reduced antifungal susceptibility by limiting drug penetration and promoting persistence [40].

Overall, the findings of this study emphasize the importance of continuous local surveillance in ICU settings, where patient complexity, antifungal exposure, and hospital ecology contribute to dynamic changes in fungal epidemiology and resistance patterns. Integration of MIC-based analysis, updated interpretive criteria such as EUCAST guidelines, and advanced statistical approaches may improve detection of emerging resistance and support more effective antifungal stewardship strategies [25,34,35,36].

## 4. Materials and Methods

### 4.1. Study Design and Data Source

This study represents a retrospective observational analysis of fungal isolates obtained from routine microbiological diagnostics. The dataset included all available fungal isolates collected between January 2021 and early 2026 in a tertiary-care hospital setting. Basic patient characteristics were collected as part of routine laboratory data, including age, sex, and hospital ward at the time of sample collection (e.g., intensive care unit). These data were used to provide a general description of the study population. Due to the retrospective laboratory-based design of the study, detailed clinical information such as comorbidities, immunosuppression status, and prior antimicrobial therapy was not consistently available.

Clinical specimens were collected at the time of ICU admission as part of routine diagnostic procedures. Only isolates with valid identification at the species level available were considered eligible for inclusion. Duplicate isolates from the same patient and same species, when identified within a short time interval, were excluded to avoid overrepresentation.

The final dataset consisted of 747 isolates derived from 725 patients, each isolate corresponding to a unique microbiological record.

Clinical specimens included respiratory samples, urine, blood and device-associated samples, sterile fluids, and wound/soft tissue samples.

Clinical data were not systematically available for all patients; therefore, differentiation between colonization and invasive infection could not be consistently established. However, all isolates included in this study were obtained from patients admitted to the intensive care unit (ICU) of a tertiary care hospital specializing in respiratory diseases. Samples were collected only from patients presenting clinical signs and symptoms suggestive of infection, in accordance with routine clinical practice. Therefore, although colonization cannot be entirely excluded, its contribution is likely limited.

### 4.2. Microbiological Identification

Samples were cultured on Sabouraud dextrose agar with chloramphenicol (PO0161A Thermo Fisher Scientific, Waltham, MA, USA) and incubated for 48 h at 35 °C, and for an additional 72 h at 30 °C. The plates were verified daily for fungal growth. From each positive sample, smears were performed and examined and at least 5 colonies were separately subcultured on chromogenic medium (Brilliance Candida agar, PO1034A; Thermo Fisher Scientific, USA) to check the purity of colonies, allowing differentiation of yeast species based on colony morphology and color characteristics. The chromogenic plates were incubated for 72 h at 30 °C and interpreted according to the producer’s recommendations. Species-level identification was performed using the Vitek 2 AES (Advanced Expert System, bioMérieux, Marcy-l’Étoile, France), following the manufacturer’s instructions. This automated system uses biochemical profiles for yeast identification. Species nomenclature was standardized across the dataset to ensure consistency in downstream analysis. Identification results were accepted based on standard confidence thresholds implemented in the system. A score of at least 96% was considered acceptable. In cases of uncertain or inconsistent results, testing was repeated to ensure reliability and for *C. auris* strains, confirmation was performed using matrix-assisted laser desorption/ionization time-of-flight mass spectrometry (MALDI-TOF MS, Bruker Daltonics, Bremen, Germany), using the manufacturer’s reference database (MBT Compass Library). Identification was interpreted according to the manufacturer’s criteria, with score values ≥ 2.0 indicating reliable species-level identification, scores between 1.7 and 1.99 indicating genus-level identification, and scores < 1.7 considered unreliable. Identified isolates were grouped into major Candida species and less frequent yeasts for further analysis.

Taxonomy follows current nomenclature; however, traditional Candida names are retained for clinical clarity.

All *C. auris* isolates were cryopreserved and are currently included in an ongoing study aimed at further characterization, including antifungal susceptibility testing using alternative methods and genetic sequencing.

Quality assurance was maintained through both internal and external control procedures. The laboratory follows a standardized internal quality control protocol and participates in external quality assessment programs conducted biannually, including testing with reference strains.

### 4.3. Antifungal Susceptibility Testing

Antifungal susceptibility testing was performed using routine laboratory methods implemented in the clinical microbiology laboratory. For each isolate, susceptibility to the following antifungal agents was recorded when available: fluconazole, voriconazole, caspofungin, micafungin, amphotericin B, and flucytosine.

Results were categorized as susceptible (S), susceptible, increased exposure (formerly known as intermediate, for better graphical representation, we will use–I), or resistant (R). The intermediate (I) category was interpreted according to EUCAST definitions as “susceptible, increased exposure,” indicating that clinical efficacy may be achieved with optimized dosing or increased drug exposure.

Interpretation of antifungal susceptibility results was primarily based on an internal laboratory protocol derived from the European Committee on Antimicrobial Susceptibility Testing (EUCAST) guidelines. For antifungal agents or species–drug combinations not covered by EUCAST breakpoints, Clinical and Laboratory Standards Institute (CLSI) criteria were selectively applied in order to provide clinically meaningful susceptibility interpretations and to support therapeutic decision-making. This approach reflects real-world clinical laboratory practice, where both EUCAST and CLSI criteria may be used depending on availability.

Non-susceptibility was defined as the combined proportion of intermediate and resistant isolates (I + R). Only isolates with valid S/I/R interpretation were included in resistance analyses. Results categorized as IE (insufficient evidence) or TRM (technical result missing) were excluded from calculations of resistance rates but were retained in the dataset for reporting of testing coverage.

Minimum inhibitory concentration (MIC) values, when available, were recorded as reported by the laboratory system. MIC50 and MIC90 values were calculated as the MIC values inhibiting 50% and 90% of isolates, respectively. MIC distributions were analyzed overall and stratified by species.

MIC values were treated as discrete measurements, and no interpolation was performed. Range values were expressed as minimum–maximum observed MICs. For exploratory analysis, MIC values were treated as continuous variables and, where appropriate, analyzed on a logarithmic scale.

*C. auris* was included in species distribution analysis but excluded from susceptibility analysis due to a lack of interpretable results.

### 4.4. Definitions

Resistance rate was defined as the proportion of isolates categorized as resistant (R) among all isolates with interpretable results (S + I + R) for a given antifungal agent.

Non-susceptibility was defined as the proportion of isolates categorized as either intermediate or resistant (I + R) among all interpretable results.

Multidrug resistance (MDR) was defined as resistance (R) to at least two antifungal agents for a given isolate. Multidrug non-susceptibility was defined as non-susceptibility (I or R) to at least two antifungal agents.

### 4.5. Data Stratification

Data were stratified according to species, year of isolation, specimen source (grouped into respiratory, urinary, mucosal/screening, sterile fluids, blood/device-related, wound/soft tissue, and other categories).

Species-level analyses were restricted to the most frequently occurring organisms in order to ensure sufficient sample size for interpretation.

### 4.6. Statistical Analysis

Descriptive statistics were used to summarize species distribution, specimen sources, and antifungal susceptibility profiles. Categorical variables were expressed as absolute numbers and percentages. Resistance and non-susceptibility rates were calculated for each antifungal agent and stratified by species and year.

Differences in susceptibility distributions across antifungal agents were evaluated using the chi-square (χ^2^) test. Effect size was estimated using Cramér’s V, with values interpreted as small (≈0.1), moderate (≈0.3), or strong (≥0.5).

To identify specific deviations from expected distributions, standardized residual analysis was performed. Residual values exceeding ±2 were considered indicative of significant deviations.

Pairwise comparisons between antifungal agents were conducted, focusing on susceptible versus resistant categories. Odds ratios (ORs) with corresponding interpretations were calculated using amphotericin B as the reference agent, due to its broad-spectrum activity and relatively stable susceptibility profile.

Temporal trends in resistance were assessed descriptively across the study period. Due to uneven yearly distribution and limited sample size in later years, formal trend modeling was not performed.

All statistical analyses were conducted using Python (version 3.11)-based statistical tools.

### 4.7. Data Handling and Limitations

The analysis was limited by variability in testing coverage across antifungal agents, as not all isolates were tested against all drugs. Additionally, the presence of non-interpretable results (IE and TRM categories) reduced the number of evaluable isolates for certain analyses.

The low number of isolates available for the year 2026 was considered insufficient for stable estimation of resistance rates and was therefore interpreted with caution in temporal analyses.

### 4.8. Ethical Considerations

This study was conducted in accordance with the ethical principles outlined in the Declaration of Helsinki. The study protocol was reviewed and approved by the Institutional Ethics Committee of the Marius Nasta Institute of Pneumology (approval no. 23343/17.10.2023). Data Access approval: 23113/12.10.2023.

Due to the retrospective observational design and the use of anonymized microbiological data obtained as part of routine clinical care, the requirement for informed consent was waived by the Ethics Committee. No identifiable patient information was accessed or included in the analysis.

All data were handled and processed in compliance with applicable national and institutional data protection regulations, ensuring confidentiality and privacy of patient information throughout the study.

## 5. Conclusions

The present study highlights substantial variability in antifungal susceptibility patterns across Candida species and antifungal agents, with a predominance of non-albicans Candida species in the analyzed cohort. Resistance rates were highest for fluconazole and flucytosine, while amphotericin B demonstrated the most preserved activity overall.

MIC analysis confirmed species-specific differences in antifungal susceptibility, particularly the reduced susceptibility of *C. glabrata* and *C. krusei* to azoles, as well as higher MIC values for echinocandins in *C. parapsilosis*. These findings reinforce the importance of accurate species identification in guiding antifungal therapy.

Statistical analysis demonstrated significant differences in susceptibility distributions across antifungal agents, while temporal analysis revealed fluctuating resistance patterns rather than a consistent increasing trend. Although multidrug resistance was relatively uncommon, a considerable proportion of isolates exhibited reduced susceptibility to multiple antifungal agents.

Taken together, these results underscore the need for continuous surveillance of antifungal resistance, integration of MIC data into routine interpretation, and the adoption of species-specific and evidence-based antifungal treatment strategies. Future studies incorporating clinical outcomes and antifungal exposure data are warranted to further elucidate the clinical impact of these findings.

## Figures and Tables

**Table 1:** Major species distribution.

Species	*n*	%
*Candida albicans*	243	32.5
*Candida glabrata*	135	18.1
*Candida parapsilosis*	84	11.2
*Candida krusei*	69	9.2
*Candida tropicalis*	62	8.3
*Candida auris*	38	5.1
*Candida lusitaniae*	31	4.1
*Candida kefyr*	25	3.3
Other species combined	60	8.0

**Table 2:** Overall antifungal susceptibility profile.

Antifungal	Tested (S + I + R)	S, *n* (%)	I, *n* (%)	R, *n* (%)	Non-Susceptibility (I + R), %
Fluconazole	385	305 (79.2)	18 (4.7)	62 (16.1)	20.8
Voriconazole	426	317 (74.4)	92 (21.6)	17 (4.0)	25.6
Caspofungin	249	194 (77.9)	45 (18.1)	10 (4.0)	22.1
Micafungin	424	297 (70.0)	116 (27.4)	11 (2.6)	30.0
Amphotericin B	588	483 (82.1)	64 (10.9)	41 (7.0)	17.9
Flucytosine	236	182 (77.1)	13 (5.5)	41 (17.4)	22.9

Antifungal susceptibility results were interpreted as susceptible (S), intermediate (I), or resistant (R) according to the breakpoints implemented in the Vitek 2 system, based on EUCAST/CLSI standards.

**Table 3:** Minimum inhibitory concentration (MIC) distributions of antifungal agents across Candida species.

Species	Antifungal Agent	MIC Range (mg/L)	MIC50	MIC90	*n*
*C. albicans*	Fluconazole	0–32	0.5	8	219
	Voriconazole	0.12–4	0.12	0.12	195
	Caspofungin	0.12–0.5	0.12	0.12	131
	Micafungin	0.06–4	0.06	0.06	215
	Amphotericin B	0.25–8	0.5	2	212
	Flucytosine	1–16	1	1	110
*C. glabrata*	Fluconazole	32–32	32	32	2 *
	Voriconazole	0.12–1	0.12	0.25	58
	Caspofungin	0.12–4	0.25	0.5	80
	Micafungin	0.06–4	0.06	0.06	111
	Amphotericin B	0.25–4	0.5	1	113
	Flucytosine	1–2	1	1	63
*C. tropicalis*	Fluconazole	0.5–32	0.5	8	55
	Voriconazole	0.12–1	0.12	0.25	53
	Caspofungin	0.12–1	0.12	0.25	38
	Micafungin	0.06–2	0.06	0.06	38
	Amphotericin B	0.25–8	0.25	0.5	53
	Flucytosine	1–16	1	1	31
*C. parapsilosis*	Fluconazole	0.5–32	0.5	8	77
	Voriconazole	0.12–4	0.12	0.12	75
	Caspofungin	0.12–1	0.5	1	50
	Micafungin	0.06–2	0.5	1	79
	Amphotericin B	0.25–4	0.5	1	77
	Flucytosine	1–1	1	1	48
*C. krusei*	Fluconazole	1–16	8	16	7 *
	Voriconazole	0.12–1	0.12	0.12	40
	Caspofungin	0.12–0.5	0.25	0.5	41
	Micafungin	0.06–0.5	0.12	0.12	42
	Amphotericin B	0.25–4	0.5	2	59
	Flucytosine	1–32	8	16	38
*C. lusitaniae*	Fluconazole	0.5–0.5	0.5	0.5	2 *
	Voriconazole	0.12–0.25	0.12	0.12	22
	Caspofungin	0.25–0.25	0.25	0.25	2 *
	Micafungin	0.12–0.5	0.12	0.12	2 *
	Amphotericin B	0.25–8	0.5	1	25
	Flucytosine	1–32	1	16	15
*C. kefyr*	Fluconazole	0.5–16	2.25	12.4	4 *
	Voriconazole	0.12–0.12	0.12	0.12	17
	Caspofungin	0.12–0.5	0.12	0.39	4 *
	Micafungin	0.06–0.5	0.12	0.39	4 *
	Amphotericin B	0.5–1	1	1	19
	Flucytosine	1–16	1	5.6	17
*C. auris*	Fluconazole	32–≥64	64	64	5 *
	Voriconazole	1	1	1	3 *
	Caspofungin	0.25	0.25	0.25	3 *
	Micafungin	0.12	0.12	0.12	5 *
	Amphotericin B	8	8	8	5 *
	Flucytosine	-	-	-	0

MIC, minimum inhibitory concentration; MIC50 and MIC90 represent the MIC values inhibiting 50% and 90% of isolates, respectively. MIC ranges are expressed in mg/L. The number of isolates tested for each antifungal agent is indicated as *n*. Values were calculated based on available MIC data for each species and antifungal agent. * Values based on small sample sizes (*n* < 10) should be interpreted with caution.

**Table 4:** Standardized residuals.

Antifungic	S	I	R
Fluconazole	0.24	−2.27	+2.36
Voriconazole	−0.25	1.45	−1.21
Caspofungin	~0	0.69	−0.93
Micafungin	−0.65	+2.59	−1.53
Amphotericin B	0.59	−1.00	−0.48
Flucytosine	~0	−1.55	+2.15

**Table 5:** Selected species-specific resistance rates (% R).

Species	Fluconazole	Voriconazole	Caspofungin	Micafungin	Amphotericin B	Flucytosine
*C. albicans*	16.5	3.8	1.2	2.3	11.1	1.5
*C. glabrata*	0.0 *	5.3	12.2	4.1	4.4	1.7
*C. parapsilosis*	15.0	8.0	0.0	1.2	5.1	0.0
*C. tropicalis*	13.0	3.8	0.0	3.2	3.7	37.5
*C. krusei*	100.0	0.0	0.0	0.0	5.1	100.0
*C. auris* **	-	-	-	0	100	-
*C. lusitaniae*	0	0	0	0	0	24
*C. kefyr*	6.2	0	0	0	0	0

* For *C. glabrata*, all 14 interpretable fluconazole results were intermediate, so resistance was 0% but non-susceptibility was 100%, ** For *C. auris*, %R was calculated only where EUCAST guidance provides interpretable criteria applicable here. Using the 5 specified isolates, micafungin resistance was 0% (5/5 MICs = 0.12 mg/L) and amphotericin B resistance was 100% (5/5 MICs = 8 mg/L). Fluconazole, voriconazole, caspofungin, and flucytosine were not reported as %R because no applicable EUCAST clinical breakpoints were available.

**Table 6:** Temporal trends in resistance rates (%) of Candida isolates to commonly used antifungal agents from 2021 to 2026.

Year	Fluconazole	Voriconazole	Caspofungin	Micafungin	Amphotericin B	Flucytosine
2021	20.3	1.3	5.6	0.0	0.6	17.2
2022	27.5	0.0	0.0	0.0	4.5	21.9
2023	6.7	8.7	5.6	1.0	12.9	10.0
2024	12.6	6.0	0.0	6.6	6.8	0.0
2025	22.4	8.6	0.0	8.3	7.9	0.0
2026	12.5	0.0	0.0	0.0	80.0	0.0

**Table 7:** Multidrug-resistant strains.

Metric	Count	Percent (%)
MDR (≥2 R)	19	2.5
Non-susceptibility ≥ 2 drugs	152	20.3

## Data Availability

The data presented in this study are available on request from the corresponding author due to privacy restrictions.

## References

[1] Pfaller M.A., Diekema D.J. (2007). Epidemiology of Invasive Candidiasis: A Persistent Public Health Problem. Clin. Microbiol. Rev..

[2] Noppè E., Eloff J.R.P., Keane S., Martin-Loeches I. (2024). A Narrative Review of Invasive Candidiasis in the Intensive Care Unit. Ther. Adv. Pulm. Crit. Care Med..

[3] Vazquez J.A., Whitaker L., Zubovskaia A. (2025). Invasive Candidiasis in the Intensive Care Unit: Where Are We Now?. J. Fungi.

[4] Arendrup M.C., Patterson T.F. (2017). Multidrug-Resistant Candida: Epidemiology, Molecular Mechanisms, and Treatment. J. Infect. Dis..

[5] Odoj K., Garlasco J., Pezzani M.D., Magnabosco C., Ortiz D., Manco F., Galia L., Foster S.K., Arieti F., Tacconelli E. (2024). Tracking Candidemia Trends and Antifungal Resistance Patterns across Europe: An In-Depth Analysis of Surveillance Systems and Surveillance Studies. J. Fungi.

[6] Calvo M., Scalia G., Trovato L. (2024). Antifungal Susceptibility Data and Epidemiological Distribution of *Candida* spp.: An In Vitro Five-Year Evaluation at University Hospital Policlinico of Catania and a Comprehensive Literature Review. Antibiotics.

[7] Beardsley J., Kim H.Y., Dao A., Kidd S., Alastruey-Izquierdo A., Sorrell T.C., Tacconelli E., Chakrabarti A., Harrison T.S., Bongomin F. (2024). *Candida glabrata* (*Nakaseomyces glabrata*): A Systematic Review of Clinical and Microbiological Data from 2011 to 2021 to Inform the World Health Organization Fungal Priority Pathogens List. Med. Mycol..

[8] Gupta A.K., Wang T. (2025). The Conundrum of Antifungal Resistance: Emergence of *Nakaseomyces glabratus* (*Candida glabrata*) in Europe with Global Implications. J. Eur. Acad. Dermatol. Venereol..

[9] Satala D., Satala G., Kulig K., Karkowska-Kuleta J., Kozik A., Rapala-Kozik M. (2025). Functional Roles of Purified Yapsins from *Candida glabrata* (*Nakaseomyces glabratus*) in Immune Modulation and Cross-Species Biofilm Formation. Sci. Rep..

[10] Gabaldón T., Martin T., Marcet-Houben M., Durrens P., Bolotin-Fukuhara M., Lespinet O., Arnaise S., Boisnard S., Aguileta G., Atanasova R. (2013). Comparative Genomics of Emerging Pathogens in the *Candida glabrata* Clade. BMC Genom..

[11] Lee Y., Robbins N., Cowen L.E. (2023). Molecular Mechanisms Governing Antifungal Drug Resistance. npj Antimicrob. Resist..

[12] Granada M., Cook E., Sherlock G., Rosenzweig F. (2024). Microbe Profile: *Candida glabrata*—A Master of Deception. Microbiology.

[13] Whaley S.G., Berkow E.L., Rybak J.M., Nishimoto A.T., Barker K.S., Rogers P.D. (2016). Azole Antifungal Resistance in Candida Albicans and Emerging Non-Albicans Candida Species. Front. Microbiol..

[14] Perlin D.S. (2015). Echinocandin Resistance in *Candida*. Clin. Infect. Dis. Off. Publ. Infect. Dis. Soc. Am..

[15] Mesa-Arango A.C., Trevijano-Contador N., Román E., Sánchez-Fresneda R., Casas C., Herrero E., Argüelles J.C., Pla J., Cuenca-Estrella M., Zaragoza O. (2014). The Production of Reactive Oxygen Species Is a Universal Action Mechanism of Amphotericin B against Pathogenic Yeasts and Contributes to the Fungicidal Effect of This Drug. Antimicrob. Agents Chemother..

[16] Delma F.Z., Melchers W.J.G., Verweij P.E., Buil J.B. (2024). Wild-Type MIC Distributions and Epidemiological Cutoff Values for 5-Flucytosine and Candida Species as Determined by EUCAST Broth Microdilution. JAC-Antimicrob. Resist..

[17] Suárez-Urquiza P., Pemán J., Gordon M., Favier P., Muñoz-Brell P., López-Hontangas J.L., Ruiz-Gaitán A. (2024). Predicting Fungemia in the ICU: Unveiling the Value of Weekly Fungal Surveillance and Yeast Colonisation Monitoring. J. Fungi.

[18] Soubani A.O. (2026). An Evolving Challenge: Fungal Infections in the Contemporary ICU. J. Fungi.

[19] Pfaller M.A., Diekema D.J. (2012). Progress in Antifungal Susceptibility Testing of *Candida* spp. by Use of Clinical and Laboratory Standards Institute Broth Microdilution Methods, 2010 to 2012. J. Clin. Microbiol..

[20] Pfaller M.A., Diekema D.J., Turnidge J.D., Castanheira M., Jones R.N. (2019). Twenty Years of the SENTRY Antifungal Surveillance Program: Results for Candida Species from 1997–2016. Open Forum Infect. Dis..

[21] Arendrup M.C. (2013). Candida and Candidaemia. Susceptibility and Epidemiology. Dan. Med. J..

[22] Preda M., Manolescu L.C.S. (2022). Romania, a Harbour of HIV-1 Subtype F1: Where Are We after 33 Years of HIV-1 Infection?. Viruses.

[23] Wu J., Jiang C., Wang H., Chen T., Chen X., Da W. (2026). The Pathogenicity and Future Treatment Strategies of *Candida albicans*. Front. Cell. Infect. Microbiol..

[24] Gómez-Gaviria M., Ramírez-Sotelo U., Mora-Montes H.M. (2023). Non-Albicans Candida Species: Immune Response, Evasion Mechanisms, and New Plant-Derived Alternative Therapies. J. Fungi.

[25] Preda M., Chivu R.D., Ditu L.M., Popescu O., Manolescu L.S.C. (2024). Pathogenesis, Prophylaxis, and Treatment of *Candida auris*. Biomedicines.

[26] Pfaller M.A., Diekema D.J., Jones R.N., Messer S.A., Hollis R.J. (2002). SENTRY Participants Group Trends in Antifungal Susceptibility of *Candida* spp. Isolated from Pediatric and Adult Patients with Bloodstream Infections: SENTRY Antimicrobial Surveillance Program, 1997 to 2000. J. Clin. Microbiol..

[27] Berkow E.L., Lockhart S.R. (2017). Fluconazole Resistance in Candida Species: A Current Perspective. Infect. Drug Resist..

[28] Manolescu L.S.C., Zugravu C., Zaharia C.N., Dumitrescu A.I., Prasacu I., Radu M.C., Letiția G.D., Nita I., Cristache C.M., Gales L.N. (2022). Barriers and Facilitators of Romanian HPV (Human Papillomavirus) Vaccination. Vaccines.

[29] Pham C.D., Iqbal N., Bolden C.B., Kuykendall R.J., Harrison L.H., Farley M.M., Schaffner W., Beldavs Z.G., Chiller T.M., Park B.J. (2014). Role of FKS Mutations in *Candida glabrata*: MIC Values, Echinocandin Resistance, and Multidrug Resistance. Antimicrob. Agents Chemother..

[30] Wiederhold N.P. (2017). Antifungal Resistance: Current Trends and Future Strategies to Combat. Infect. Drug Resist..

[31] Hui S.T., Gifford H., Rhodes J. (2024). Emerging Antifungal Resistance in Fungal Pathogens. Curr. Clin. Microbiol. Rep..

[32] Pappas P.G., Kauffman C.A., Andes D.R., Clancy C.J., Marr K.A., Ostrosky-Zeichner L., Reboli A.C., Schuster M.G., Vazquez J.A., Walsh T.J. (2016). Clinical Practice Guideline for the Management of Candidiasis: 2016 Update by the Infectious Diseases Society of America. Clin. Infect. Dis. Off. Publ. Infect. Dis. Soc. Am..

[33] Kullberg B.J., Arendrup M.C. (2015). Invasive Candidiasis. N. Engl. J. Med..

[34] European Committee on Antimicrobial Susceptibility Testing (2026). Breakpoint Tables for Interpretation of MICs for Antifungal Agents.

[35] Arendrup M.C. (2014). Update on Antifungal Resistance in *Aspergillus* and *Candida*. Clin. Microbiol. Infect. Off. Publ. Eur. Soc. Clin. Microbiol. Infect. Dis..

[36] Cernat E.M., Dima A., Popescu C., Neagu A., Betianu C., Moga M., Manolescu L.S.C., Barbilian A. (2024). Anterior Intercondylar Notch Geometry in Relation to the Native Anterior Cruciate Ligament Size. J. Clin. Med..

[37] Roberts J.A., Sime F.B., Lipman J., Hernández-Mitre M.P., Baptista J.P., Brüggemann R.J., Darvall J., De Waele J.J., Dimopoulos G., Lefrant J.-Y. (2025). Are Contemporary Antifungal Doses Sufficient for Critically Ill Patients? Outcomes from an International, Multicenter Pharmacokinetics Study for Screening Antifungal Exposure in Intensive Care Units—The SAFE-ICU Study. Intensive Care Med..

[38] Garnacho-Montero J., Barrero-García I., León-Moya C. (2024). Fungal Infections in Immunocompromised Critically Ill Patients. J. Intensive Med..

[39] Hernandez-Reyes F.B., Muñoz-Miranda L.A., Kirchmayr M.R., Ortiz-Lazareno P.C., Cortés-Zárate R., Iñiguez-Moreno M., Jacobo-Cuevas H., Nava-Valdivia C.A. (2025). Antifungal Susceptibility and Resistance-Associated Gene Expression in Nosocomial Candida Isolates. J. Fungi.

[40] Hossain C.M., Ryan L.K., Gera M., Choudhuri S., Lyle N., Ali K.A., Diamond G. (2022). Antifungals and Drug Resistance. Encyclopedia.

